# Correction: A crystalline sponge based on dispersive forces suitable for X-ray structure determination of included molecular guests

**DOI:** 10.1039/c6sc90013e

**Published:** 2016-02-09

**Authors:** Elena Sanna, Eduardo C. Escudero-Adán, Antonio Bauzá, Pablo Ballester, Antonio Frontera, Carmen Rotger, Antonio Costa

**Affiliations:** a Departament de Química , Universitat de les Illes Balears , Palma , 07122 , Spain . Email: antoni.costa@uib.es ; Fax: +34 971 172436 ; Tel: +34 971 173266; b Institut of Chemical Research of Catalonia (ICIQ) , Avda. Països Catalans 16 , 43007 Tarragona , Spain; c Catalan Institution for Research and Advanced Studies (ICREA) , Passeig Lluis Companys 23 , 08010 , Barcelona , Spain

## Abstract

Correction for ‘A crystalline sponge based on dispersive forces suitable for X-ray structure determination of included molecular guests’ by Elena Sanna *et al.*, *Chem. Sci.*, 2015, **6**, 5466–5472.



## 

The authors regret that in the original article incorrect CCDC numbers were presented for the 11 crystal structures discussed therein, these errors are found in the caption to Fig. 5 and the ESI footnote. In both cases, ‘CCDC codes 1012389–1012397 and 1063714’ should have read as ‘CCDC codes 1012389, 1012391–1012399 and 1063714’. The CCDC files within the ESI of the original article have also been updated accordingly.

The Royal Society of Chemistry apologises for these errors and any consequent inconvenience to authors and readers.

